# Establishment of a rapid detection method for severe fever with thrombocytopenia syndrome virus based on reverse transcription-recombinase polymerase amplification with lateral flow nucleic acid chromatography assay technology

**DOI:** 10.3389/fmicb.2026.1934648

**Published:** 2026-09-09

**Authors:** Zhoujie Ma, Yongchuan Gan, Qiyun Liang, Lufei Yuan, Lele Song, Rong Jiang, Jilu Shen

**Affiliations:** 1 Department of Clinical Laboratory, The First Affiliated Hospital of Anhui Medical University, Hefei, China; 2 Department of Clinical Laboratory, Anhui Public Health Clinical Center, Hefei, China; 3 Shenzhen Tailored Medical Ltd., Shenzhen, China

**Keywords:** detection, LFNA, qPCR, RT-RPA, SFTSV

## Abstract

**Introduction:**

Severe fever with thrombocytopenia syndrome virus (SFTSV), a novel bunyavirus associated with febrile illness, thrombocytopenia, and leukopenia, represents a considerable public health threat.

**Methods:**

In this study, we developed a visual detection platform for SFTSV by integrating reverse transcription-recombinase polymerase amplification (RT-RPA) with lateral flow nucleic acid chromatography assay (LFNA). The method operates under isothermal conditions, and delivers results within 40 minutes. Using a conserved region of the S segment as the amplification target.

**Results:**

The assay demonstrated a detection limit of 10^1^ copies/μL and showed no cross-reactivity with 12 other common clinical pathogens or 6 less common clinical pathogens. An evaluation of 80 SFTSV-positive clinical samples and 16 SFTSV-negative clinical samples showed that, compared with quantitative PCR (qPCR), the method demonstrated excellent sensitivity and specificity.

**Discussion:**

The RT-RPA-LFNA platform is easy to operate, provides rapid results and is cost-effective, and therefore has potential for use in point-of-care testing (POCT), particularly in resource-limited primary healthcare settings. This approach not only provides a reliable tool for SFTSV diagnosis but also holds potential for the detection of other pathogens.

## Introduction

Severe fever with thrombocytopenia syndrome virus (SFTSV), a novel bunyavirus, is a single-stranded negative-sense RNA virus characterized by clinical manifestations including fever, gastrointestinal symptoms, thrombocytopenia, and leukopenia (Prayitno et al., 2025; Bryden et al., 2022). Its genome comprises three segments: the L segment encoding RNA-dependent RNA polymerase (RdRp), the M segment encoding glycoproteins Gn and Gc, and the S segment encoding nucleoprotein (Np) and non-structural protein (Ns) (Park et al., 2023; Bratuleanu et al., 2023). While all three segments harbor conserved regions enabling nucleic acid detection—with no significant differences in sensitivity or specificity among them—the S segment is prioritized for its higher conservation and superior detection efficiency in clinical serum samples (Lee et al., 2020; Li A. et al., 2021; Zhuang et al., 2018).

SFTSV was first identified in 2009 in rural areas of Hubei and Henan provinces in China. It is an emerging tick-borne pathogen, with the Asian longhorned tick (*Haemaphysalis longicornis*) serving as its primary vector (Gao et al., 2023; Cui et al., 2024). The disease has since become endemic in seven central and eastern Chinese provinces, with Anhui Province particularly affected (Wang et al., 2015; Chen et al., 2022). People usually become infected with SFTSV through the bite of an infected tick or contact with infected animals. In addition, the virus can also spread through contact with a patient’s blood, leading to possible transmission within families and in healthcare settings (Wen et al., 2024). Compounded by the lack of licensed vaccines or specific therapeutics (Reece et al., 2018), early and accurate SFTSV detection is critical for mitigating transmission and improving patient outcomes (Li J. et al., 2021).

Current SFTSV detection methods fall into three main categories: nucleic acid testing (Li et al., 2020), serological assays (Jiao et al., 2012), and virus isolation (Goldsmith et al., 2013). Serological methods such as IgM-capture ELISA and indirect IgG ELISA are cost-effective but prone to cross-reactivity, limiting their specificity (Chang et al., 2025; Varghese et al., 2023). Virus isolation, while highly accurate, is time-intensive and impractical for rapid clinical diagnosis (Zhang et al., 2022). Nucleic acid-based techniques have thus become the mainstay of molecular diagnosis, with polymerase chain reaction (PCR) being the most widely used since its invention in the 1980s (Tan et al., 2022). However, PCR relies on precise temperature cycling equipment, specialized operation, and prolonged turnaround times, restricting its utility for on-site testing (Fan et al., 2025). Although quantitative PCR (qPCR) has evolved as the gold standard for SFTSV detection, it remains constrained by the need for trained personnel and sophisticated laboratory infrastructure (Sun et al., 2012; Wang et al., 2014).

Recombinase Polymerase Amplification (RPA) (Piepenburg et al., 2006) has emerged as a promising isothermal amplification technology for point-of-care testing (POCT). RPA primarily consists of a recombinase, a polymerase, and a single-strand-binding protein. First, the recombinase binds to the primer to form a complex and searches for homologous sequences in the target double-stranded DNA. The recombinase then uses its strand-displacement activity to facilitate the insertion of the primer into the corresponding site. Following binding, the single-strand binding protein stabilizes the displaced DNA, while the strand-displacement DNA polymerase extends the primer from the 3′ end, thereby enabling exponential amplification of the DNA. Furthermore, operating at 37–40 °C, RPA enables rapid target amplification within 30 min, requires minimal instrumentation, and features straightforward primer design (Piepenburg et al., 2006; Wu et al., 2023; Tan et al., 2022; Zhu et al., 2022)—addressing key limitations of alternative isothermal methods such as Loop-mediated Isothermal Amplification [LAMP, which suffers from complex primer design and non-specific amplification (Wei et al., 2022)] and Nucleic Acid Sequence-Based Amplification [NASBA, with ~2-h reaction times (Prajapati et al., 2022)]. Complementing RPA’s advantages, Lateral Flow Immunoassay (LFIA) integrates chromatographic principles with antigen–antibody specificity to deliver visual, low-cost, and user-friendly results, making it ideal for resource-limited settings (Majdinasab et al., 2023; Lee et al., 2025).

Previous methods for detecting SFTSV at the molecular level have included LAMP and qPCR, however, we employed RT-RPA for amplification, which offers the advantages of simple primer design and a short amplification time. Furthermore, by incorporating a reverse transcriptase into the RPA protocol, we were able to amplify RNA targets. Based on this, we developed and validated an integrated RT-RPA-LFNA platform for the rapid, visual detection of SFTSV. This method targets the conserved S segment, combines the rapid isothermal amplification of RT-RPA with the simple visual readout of LFNA, and completes detection within 40 min. Our key innovation lies in the design of specific nucleic acid tags to modify the primers, and the design of corresponding nucleic acid probes to label the test strips, thereby enhancing the sensitivity of our assay to a level comparable to qPCR, whilst achieving a detection time significantly shorter than that of qPCR. We evaluated its analytical sensitivity, specificity, and clinical performance using archived patient samples, demonstrating its potential as a robust, user-friendly tool for SFTSV diagnosis in primary care and field settings.

## Materials and methods

### Reagents and chemicals

Colloid gold-labeled ssDNA probes, T-line detection ssDNA probes, C-line detection ssDNA probes, primers, the SFTSV cloning vector (10^10^ copies/μL) and the cloning vectors for pathogens phylogenetically related to SFTSV, or pathogens that exhibit symptoms similar to those of SFTSV infection (Hartland virus, Hunter Island virus, Hantavirus, *Ehrlichia chaffeensis*, *Anaplasma phagocytophilum*, and *Orientia tsutsugamushi*) were synthesized by Sangon Biotech (Shanghai, China). The RT-RPA kit (WLRB8207KIT) was provided by Wobo Biotechnology (Nanjing, China). The RT-RPA product purification kit (AE0101-C) was supplied by Sparkjade (Shandong, China). The nucleic acid extraction kit (DA034) was provided by DAANGENE (Guangzhou, China). The SFTSV nucleic acid detection kit (PCR-fluorescent probe method, DA0340) was provided by DAANGENE (Guangzhou, China).

### Epidemiological analysis

The data on all 237 patients diagnosed with SFTSV at the First Affiliated Hospital of Anhui Medical University in 2025 were compiled into charts illustrating the relationship between patient numbers and months, the geographical distribution of patients, the gender distribution of patients, and the age distribution of patients. These statistical analysis charts were all created using Excel.

### Nucleic acid extraction

All nucleic acids used for detection were extracted according to the instructions in the nucleic acid extraction kit (DAANGENE, Guangzhou). Brief procedure: add protease K, the sample and the viral lysate containing carrier RNA to a centrifuge tube, centrifuge at high speed, then incubate at 72 °C. Treat sequentially with anhydrous ethanol, inhibitor removal buffer and deionised water. Finally, elute the target nucleic acid using the elution buffer. The resulting nucleic acid is isolated RNA. After nucleic acid extraction, the concentration and purity of the nucleic acids were measured using OneDrop Spectrophotometer. All nucleic acids are stored at −80 °C.

### Design of RT-RPA primers for SFTSV

Although the L, M, and S segments of SFTSV can all be used for nucleic acid detection, studies have shown that the sequence of the S segment is the most conserved and has the highest detection rate in patient serum samples. Therefore, the S segment is the preferred target for viral nucleic acid detection (Huang et al., 2022; Vogel et al., 2020). Multiple published SFTSV S-segment genomic sequences were retrieved and downloaded from the NCBI database (GenBank version numbers: PV774076.1-PV774086.1). SnapGene software was used for multiple sequence alignment to identify conserved gene sequences of SFTSV. Based on these conserved sequences, multiple pairs of RT-RPA primers were designed using Primer Premier 5 software, and the specificity of the primers was verified using the NCBI-BLAST online tool.

Primer design software parameters: Tm value set between 50 °C and 55 °C; primer length between 25 bp and 35 bp; amplified product length set between 100 bp and 200 bp; GC content set between 40 and 60%.

All other parameters were kept at default settings. Multiple primer pairs were selected based on the software-assigned scores. The specific primer sequences are listed in Table 1. The synthesized primers are stored at −20 °C.

**Table 1:** RT-RPA primers sequences.

Primers	Sequence (5′-3′)
SFTSV-F1	TGTTCTTCTCCATCAAGAACAGCTGGGCAATGGAA
SFTSV-R1	AAGTCTGAGCCTTCGCTTCTCTATGGCTTCAAGAG
SFTSV-F2	GCCATAGAGAAGCGAAGGCTCAGACT
SFTSV-R2	GCTTACGCAATGAGGAAGAAGTGAAT
SFTSV-F3	ACTACAAGGACATGAGGTGCTACTCCCAGAGA
SFTSV-R3	CCATTGCCCAGCTGTTCTTGATGGAGAAGAAC
SFTSV-F4	CCATAGAGAAGCGAAGGCTCAGACTTGGGTTA
SFTSV-R4	CGCAATGAGGAAGAAGTGAATAAGTGGTGGTT
SFTSV-F5	CTAATAACTGGACTATCCCCAATTCTGGATGTG
SFTSV-R5	AACAGTCTAACAGAGGCTTACGCAATGAGGAAG
SFTSV-F6	ATTGAGCTGCTGCTCACCAAACTCCACTGCAA
SFTSV-R6	CATCAAGAAGCTGAAGGAGACAGGTGGAGATG
SFTSV-F7	AAGTATCTTTTTGAAGGGGACATGA
SFTSV-R7	GAGGAAGAAGTGAATAAGTGGTGGT
SFTSV-F8	ACAGGATAACAAAAGGCCAAAAGTC
SFTSV-R8	ACGCAATGAGGAAGAAGTGAATAAG

### Purification of amplified target fragments and primers screening

In a 50 μL RT-RPA reaction system, the following components were included: 29.4 μL of Rehydration Buffer (A buffer), 2 μL of forward primer (2 μM), 2 μL of reverse primer (2 μM), 5 μL of nucleic acid template, 9.1 μL of nuclease-free water (DEPC-treated water) and 2.5 μL of 280 mM magnesium acetate solution (B buffer). The above components were added sequentially to the reaction tube containing the enzyme components (which contains reverse transcriptase), following the instructions for the RT-RPA Kit (Wobo, Nanjing). Subsequently, 80 μL of liquid paraffin oil was added to prevent aerosol contamination during subsequent lid-opening procedures. Finally, the reaction tube was placed in a metal bath (a dry bath containing metal beads) and incubated at 37 °C for 30 min. The specific reagent volumes added are detailed in Table 2. DEPC-treated water is stored at 4 °C, and all other RT-RPA reaction reagents are stored at −20 °C.

**Table 2:** Reaction components for RT-RPA amplification.

Component	Volume
A buffer	29.4 μL
forward primer (2 μM)	2 μL
reverse primer (2 μM)	2 μL
nucleic acid template	5 μL
DEPC-treated water	9.1 μL
B buffer	2.5 μL
Total volume	50 μL

The RT-RPA-amplified product was purified according to the instructions in the RT-RPA product purification kit (Sparkjade, Shandong), and the purified product was analyzed by 2% agarose gel electrophoresis to identify the optimal primer pair. The RT-RPA product purification kit is stored at room temperature.

### Preparation of lateral flow nucleic acid assay (LFNA) test strips

Following the established fabrication method described in the literature (Qin et al., 2021), the coupling pad, sample pad, nitrocellulose (NC) membrane, and absorbent pad were synthesized and assembled onto a backing card to prepare a lateral flow nucleic acid (LFNA) test strip. After primers screening, the 5′ ends of the forward and reverse primers will each be labeled with a specific and distinct nucleic acid tags (NAT), and C12 spacer will be inserted to prevent NAT polymerization during amplification. The 3′ end of the recognition probe (RP) was functionalized with a thiol group and conjugated to gold nanoparticles to serve as a gold nanoparticle-conjugated probe (GNP-RP). The 3′ ends of the capture probe (CP) and the control capture probe (CCP) are biotinylated and are used to generate the test line (T Line) and control line (C Line), respectively. The specific sequences are listed in Tables 3, 4. The test strips are stored at room temperature in a dry environment, and all probes are stored at −20 °C.

**Table 3:** Sequences of LFNA probes.

Probes	Sequence (5′-3′)
Gold nanoparticle-conjugated probes (GNP-RP)	TGTTTGTTTGTTTGTTTTTTTT-SH
Test line probes (CP)	GCTAGTGCTCGATCAGTTTTTT-Biotin
Control line probes (CCP)	AACAAACAAACAAACA-Biotin

**Table 4:** Sequences of LFNA modified primers.

Primes	Sequence (5′-3′)
NAT-SFTSV-F	AACAAACAACAAACA/iSpC12/GCCATAGAGAAGCGAAGGCTCAGACT
NAT-SFTSV-R	CTGATCGAGCACTAGC/iSpC12/GCTTACGCAATGAGGAAGAAGTGAAT

### Establishment of the RT-RPA-LFNA detection method

**Figure 1:**
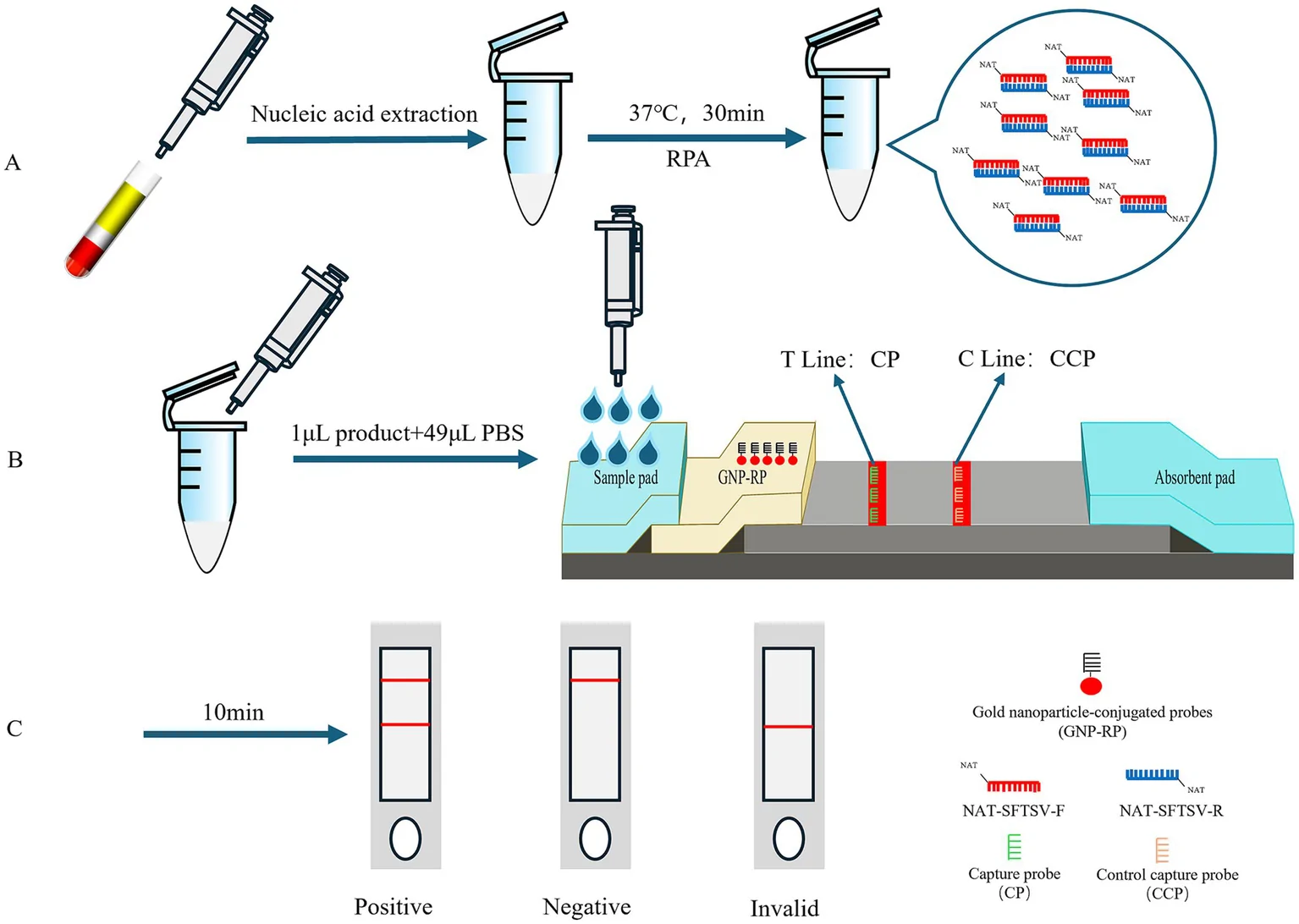
RT-RPA-LFNA testing process. **(A)** The prepared RT-RPA system is incubated at 37 °C for 30 min to amplify the sample. **(B)** One microliter of the amplified product is mixed with 49 μL of PBS in a new centrifuge tube, and then the entire mixture is pipetted onto the sample pad. **(C)** The test result is determined by observing the color development on the test strip. The positive result is indicated by color development in both the T line and the C line. The negative result is indicated by color development only in the C line. The invalid result is indicated by no color development in the C line. rRT-RPA: Reverse transcription-recombinase polymerase amplification. NAT: Nucleic acid tags, CP: Capture probe, CCP: Control capture probe. GNP-RP: Gold nanoparticle-conjugated probe.

One microliter of the RT-RPA amplification product and 49 μL of PBS buffer are added to a new centrifuge tube and mixed thoroughly. The mixture is then applied to the sample pad of the test strip. Since GNP-RP binds specifically to the NAT of the forward primer and to CCP, and the NAT of the reverse primer binds specifically to CP, when the sample is positive, the nucleic acid in the sample is amplified by the forward and reverse primers, producing two specific NAT fragments. As the products migrate, they sequentially bind to GNP-RP and CP, causing the T line to develop. Subsequently, excess GNP-RP binds to CCP, causing the C line to develop. When the sample is negative, due to the lack of bridging provided by the amplification products, the GNP probe cannot bind to the T line, and excess GNP-RP can only bind to CCP, causing the C line to develop. A specific flowchart is provided in Figure 1.

### Limit of detection (LOD) determination

The standard SFTSV cloning vector was prepared by inserting the target gene sequence of the SFTSV S fragment into the pUC57 vector (synthesized by Sangon Shanghai). The stock solution of the standard SFTSV cloning vector (10^10^ copies/μL) was prepared based on the fragment size of the vector and the reagent instructions. It was then serially diluted 10-fold with DEPC-treated water to prepare nine standard samples at different concentrations: 10^8^, 10^7^, 10^6^, 10^5^, 10^4^, 10^3^, 10^2^, 10^1^, and 10^0^ copies/μL. These standard samples at various concentrations were tested using the aforementioned RT-RPA-LFNA detection method. To quantitatively assess the sensitivity of this method, the test line (T line) and control line (C line) on the test strips were subjected to quantitative grayscale analysis using ImageJ software. A comparison graph was created in Excel, with the vertical axis showing the ratio of the gray-scale values of the T line to those of the C line, and the horizontal axis showing sample concentration. Based on the results of repeated experiments, we have defined the limit of detection (LOD) as the minimum number of copies for which the test strip displays a positive result and the ratio of the grey scale values of the T line to the C line is greater than 0.1. These standards were also analyzed by qPCR (Thermo Fisher Scientific 7,500 Real-Time PCR System) to compare their sensitivity with the gold standard. The stock solution of the standard SFTSV cloning vector is stored at −20 °C, other standard solutions of different concentrations are prepared as needed.

### Determination of the LOD for the gold standard qPCR

The cycling conditions for the 7,500 Real-Time PCR System were set according to the instructions for the SFTSV nucleic acid detection kit (PCR-fluorescent probe method, DAANGENE, Guangzhou) as follows: 15 min at 50 °C for one cycle; 15 min at 95 °C for one cycle; and 45 cycles of 94 °C for 15 s to 55 °C for 45 s (fluorescence detection). The detection reagents and the aforementioned standard samples were then added to the reaction tubes in sequence according to the instructions. If the Ct value is greater than 35 or “Undetermined,” it indicates that the viral concentration is below the detection limit, and the result is reported as negative. For quantitative testing, results are displayed in TCID_50_/mL. The conversion between TCID_50_ and copies is as follows: 10^x^TCID_50_/mL = 0.496 × 10^x^copies/μL. All qPCR assay kits are stored at −20 °C.

### Cross-reactivity evaluation

Twelve common pathogens in clinical molecular laboratories: Influenza A virus (throat swab), Influenza B virus (throat swab), SARS-CoV-2 (throat swab), Hepatitis B virus (serum), Norovirus (feces), Adenovirus (throat swab), Respiratory Syncytial Virus (throat swab), *Mycoplasma pneumoniae* (serum), Epstein–Barr virus (whole blood), Rotavirus (feces), *Mycobacterium tuberculosis* (sputum), and *Helicobacter pylori* (saliva) were tested using RT-RPA-LFNA to validate the method’s specificity for SFTSV, prevent cross-contamination between these common pathogens and SFTSV from affecting test results, and avoid non-specific amplification of these pathogens’ nucleic acids with the primers selected for this platform. The clinical samples are all stored at −80 °C.

Since other pathogens associated with SFTSV phylogenetics or showing symptoms similar to SFTSV infection (Heartland virus, Hunter Island virus, Hantavirus, *Ehrlichia chaffeensis*, *Anaplasma phagocytophilum*, *Orientia tsutsugamushi*) are clinically rare and no clinical samples have been collected in this region, sequences of these pathogens were downloaded from the NCBI database (GenBank: LC629153.1, NC_078066.1, PX445904.1, PX682657.1, PZ302318.1, PX991847.1) and synthesized corresponding cloning vectors to validate cross-reactivity. All cloning vectors were uniformly diluted to 10^8^ copies/μL for the experiment.

### Collection and evaluation of clinical samples

All 96 clinical serum samples (80 SFTSV-positive samples and 16 SFTSV-negative samples) were collected from the Anhui Provincial Public Health Clinical Center (North Campus of the First Affiliated Hospital of Anhui Medical University). All samples were obtained from hospitalized patients and were confirmed as SFTSV-positive or SFTSV-negative by qPCR results from the molecular laboratory. Epidemiological information for the 80 clinical positive cases is provided in Supplementary Table S1. The collected serum samples were processed using the aforementioned nucleic acid extraction method and subsequently tested using the RT-RPA-LFNA assay. All clinical samples are stored at −80 °C.

After nucleic acid extraction from all clinically positive samples, the concentration and purity of positive samples were measured using OneDrop Spectrophotometer. The specific results are provided in Supplementary Table S2.

Specificity was measured as [100 × number true negative (TN)/number TN + number false positive (FP)], and sensitivity was evaluated as [100 × number true positive (TP)/number TP + number false negative (FN)].

## Results

### Epidemiological analysis of SFTSV

**Figure 2:**
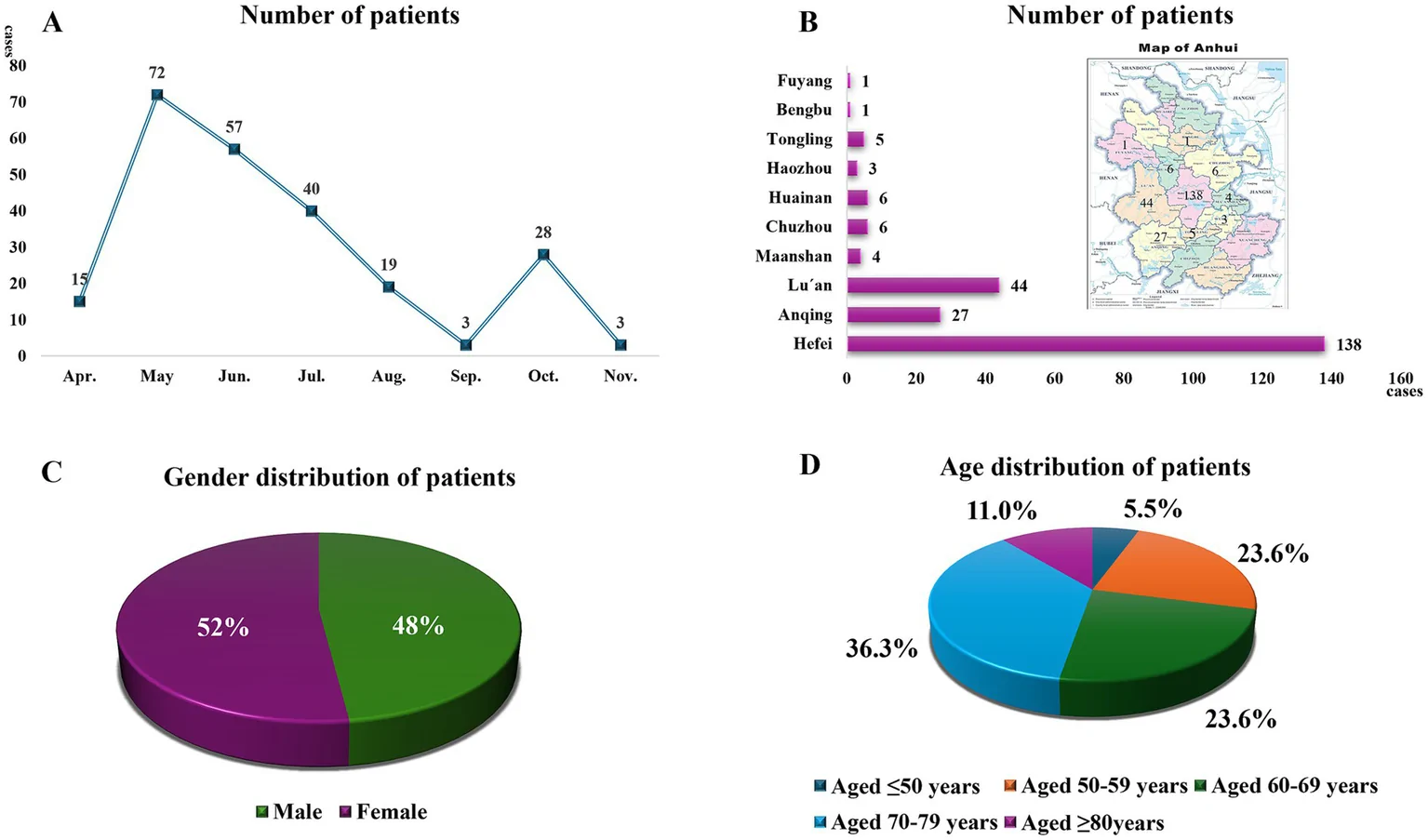
Epidemiological analysis results of SFTSV. **(A)** Distribution of SFTSV cases by month. **(B)** Geographical distribution of SFTSV cases. **(C)** Gender distribution of SFTSV patients. **(D)** Age distribution of SFTSV patients.

In this study, we focused on patients diagnosed with SFTSV (237 cases) at the First Affiliated Hospital of Anhui Medical University in 2025. Statistical analyses were conducted on the relationship between patient numbers and months, geographical distribution, as well as patient age and gender distribution. The results indicated that SFTSV enters an epidemic period from April to November, with cases primarily concentrated in May (72 cases), June (57 cases), July (40 cases), and October (28 cases) (Figure 2A). SFTSV cases were reported in various cities across Anhui Province, mainly focusing on cities rich in mountainous resources such as Hefei (138 cases), Lu′an (44 cases), and Anqing (27 cases) (Figure 2B). Most patients contracted the disease due to tick bites while engaging in agricultural work in mountainous areas. The gender distribution among patients showed little difference, with males accounting for 48% (114 cases) and females for 52% (123 cases) (Figure 2C). The disease was distributed across all age groups, primarily concentrated in the 50–59 age group (56 cases), 60–69 age group (56 cases), and 70–79 age group (86 cases) (Figure 2D). As a high-risk region, Anhui Province shares many similarities with the national epidemiological characteristics of SFTSV. The incidence peaks from May to July, predominantly affects middle-aged and elderly individuals, and most patients are farmers or forestry workers from rural mountainous and hilly areas (Chen et al., 2022). Therefore, the prevalence of SFTSV within Anhui Province remains relatively severe, highlighting the importance of achieving rapid, sensitive, and low-cost detection of SFTSV in the early stages of infection. The specific experimental results are shown in Figure 2.

### Screening of RT-RPA primers

In this study, we used nucleic acids extracted from the serum of novel bunyavirus-positive patients as experimental templates. We conducted RT-RPA experiments with different primers designed by our team, purified the amplification products, and subjected them to agarose gel electrophoresis for comparative analysis to evaluate amplification efficiency. The optimal RT-RPA primers were selected and validated using multiple samples. Following confirmation, experiments were also conducted with modified versions of these primers.

**Figure 3:**
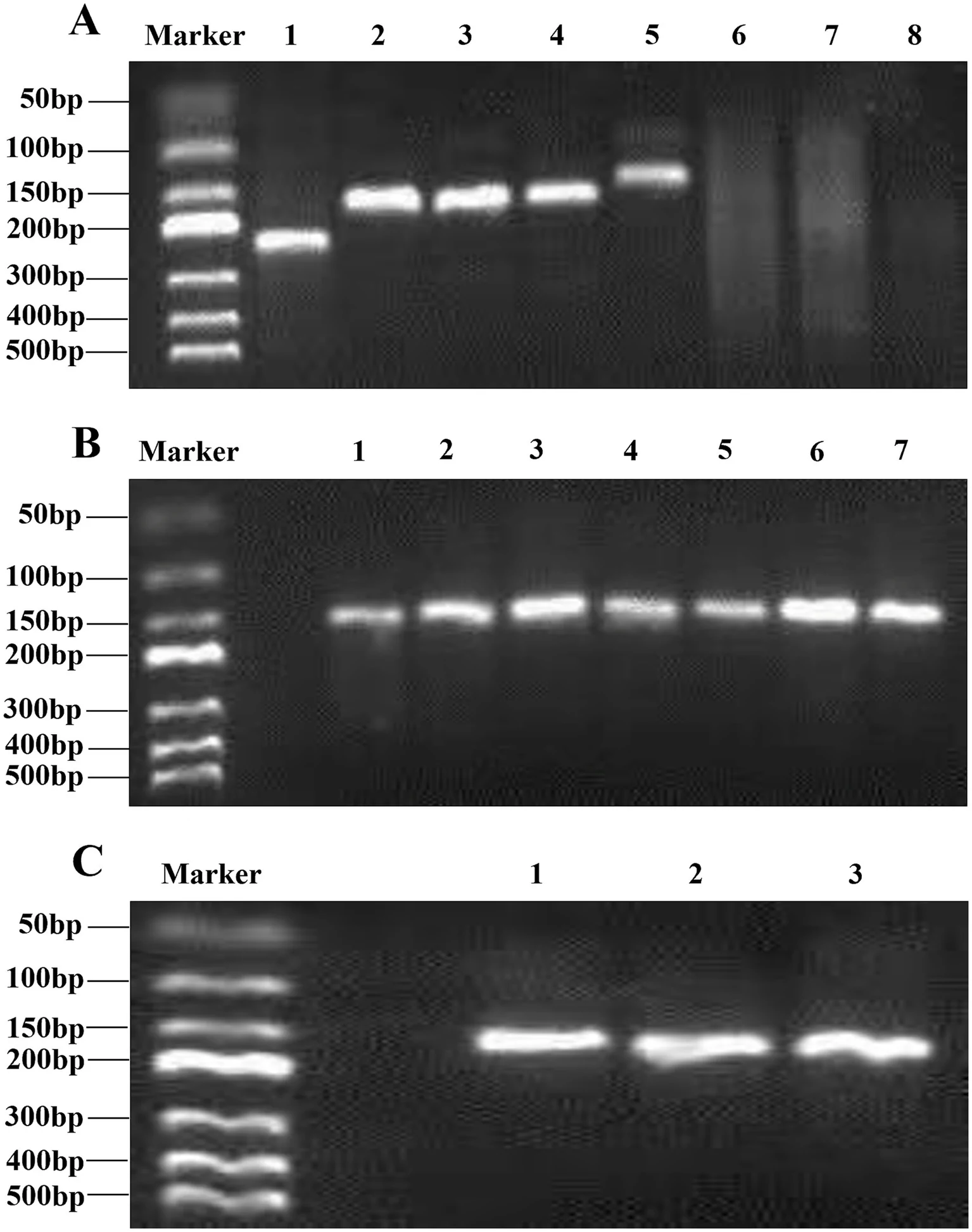
RT-RPA primers screening results. **(A)** Primer 1 represents F1 + R1, Primer 2 represents F2 + R2, Primer 3 represents F3 + R3, Primer 4 represents F4 + R4, Primer 5 represents F5 + R5, Primer 6 represents F6 + R6, Primer 7 represents F7 + R7, Primer 8 represents F8 + R8. **(B)** Multiple samples were randomly selected for further validation of Primer 2. **(C)** Validation of the modified version of Primer 2.

The experimental results showed that most of the designed primers successfully achieved specific amplification of the template, producing the target bands. By comparing the electrophoresis results of different primers, we evaluated the outcomes based on band intensity and the presence or absence of primer dimers, ultimately selecting Primer 2 as the optimal RT-RPA primer (Figure 3A). Subsequently, multiple positive samples were randomly selected for further validation of this primer (Figure 3B), and the modified version of this primer was also tested (Figure 3C). This primer demonstrated excellent amplification efficiency and specificity during amplification, providing a reliable foundation for subsequent experiments.

### Evaluation of the LOD for the RT-RPA-LFNA method

**Figure 4:**
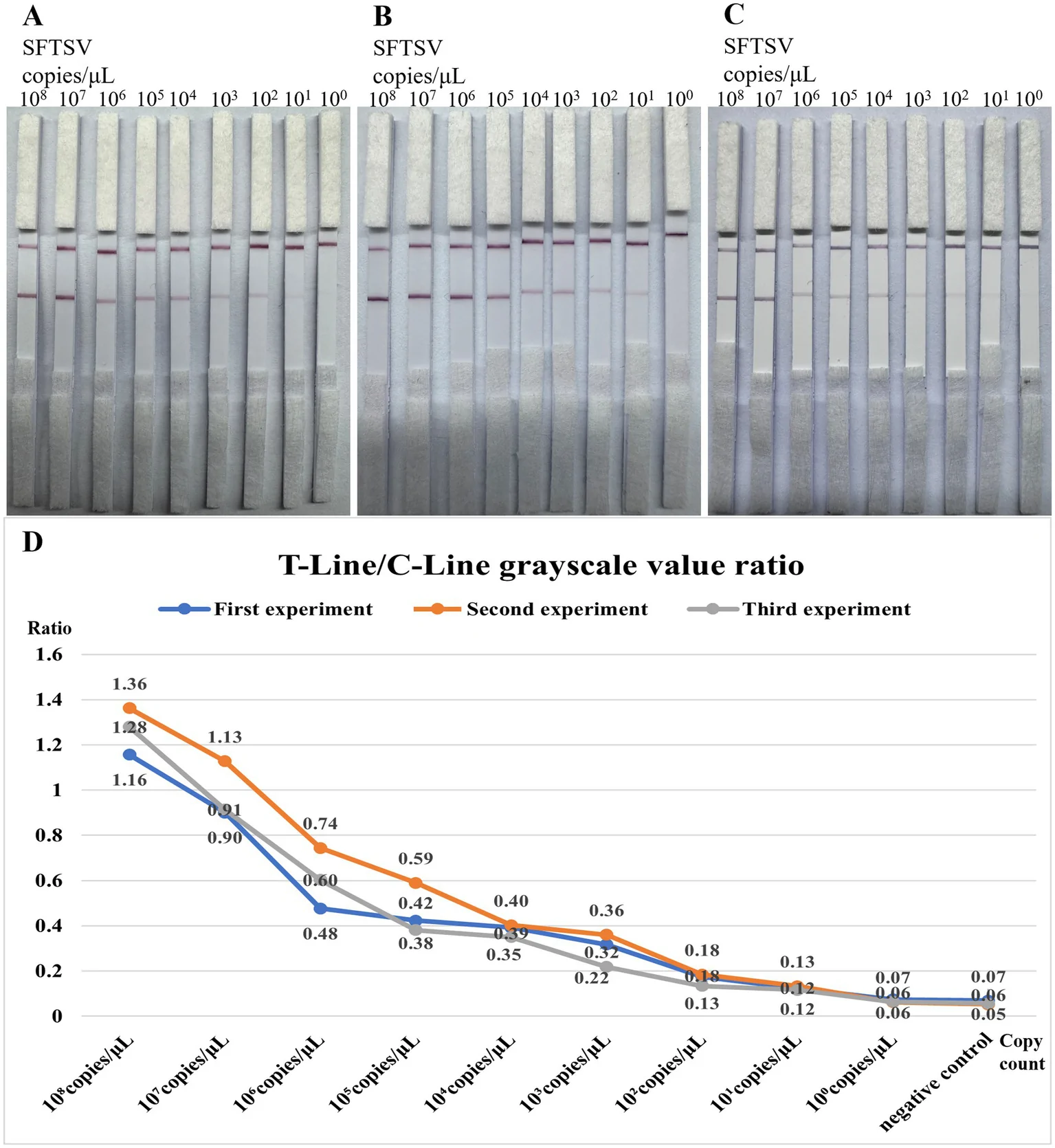
RT-RPA-LFNA LOD evaluation result. **(A)** Results from the first replicate experiment, the concentrations from left to right are 10^8^, 10^7^, 10^6^, 10^5^, 10^4^, 10^3^, 10^2^, 10^1^, and 10^0^ copies/μL. At a concentration of 10^1^ copies/μL, the test result still showed a faint positive, but at 10^0^ copies/μL, the result was negative. **(B)** Results from the second replicate experiment. **(C)** Results from the third replicate experiment. **(D)** The ratio results showed a decreasing trend with declining sample concentration and approximated that of the negative control when the concentration was 10^1^ copies/μL.

In this study, we used the constructed SFTSV cloning vector as the base sample and performed gradient dilutions to prepare nine plasmid samples with concentrations of 10^8^, 10^7^, 10^6^, 10^5^, 10^4^, 10^3^, 10^2^, 10^1^, and 10^0^ copies/μL. These samples were tested using the RPA-LFNA detection method. A total of three repeatability tests were conducted. Results showed test line color intensity decreased with sample concentration, with negative results at 10^0^ copies/μL—confirming a SFTSV detection limit (LOD) of 10^1^ copies/μL, which is comparable to the sensitivity of the gold-standard qPCR (Figures 4A–C). This level of detection limit indicates that the platform can effectively detect the target pathogen nucleic acid, holding significant importance for early diagnosis and precision medicine. The statistical results showed that the ratio of the T line grayscale value to the C line grayscale value exhibited a decreasing trend as the concentration of the samples declined. Furthermore, when the sample concentration was 10^1^ copies/μL, the ratio approximated that of the negative control and greater than 0.1, providing additional evidence that the LOD for this method is 10^1^ copies/μL (Figure 4D). The specific grayscale values can be found in Supplementary Table S3.

### Evaluation of the LOD for qPCR (gold standard)

To compare the sensitivity difference between RT-RPA-LFNA and the gold standard, we detected the standard SFTSV cloning vector using qPCR. Samples with concentrations of 10^4^ copies/μL, 10^3^ copies/μL, 10^2^ copies/μL, 10^1^ copies/μL, 10^0^ copies/μL, 10^−1^ copies/μL, along with negative and positive controls, were subjected to qPCR. The results showed that when the sample concentration was 10^0^ copies/μL, the Ct value was 36.98 (a Ct value > 35 is considered negative). Additionally, the results for both the negative and positive controls were normal, indicating that the LOD of qPCR is 10^1^ copies/μL, which is comparable to the LOD of RT-RPA-LFNA. The specific experimental results are shown in Table 5.

**Table 5:** qPCR LOD evaluation results.

Samples	Ct value	Results (Ct value > 35 is considered negative)
10^4^ copies/μL	22.96	(+)
10^3^ copies/μL	26.28	(+)
10^2^ copies/μL	29.89	(+)
10^1^ copies/μL	33.44	(+)
10^0^ copies/μL	36.98	(−)
10^−1^ copies/μL	Undetermined	(−)
Negative control	Undetermined	(−)
Positive control	22.83	(+)

### Cross-reactivity evaluation of the RT-RPA-LFNA method

**Figure 5:**
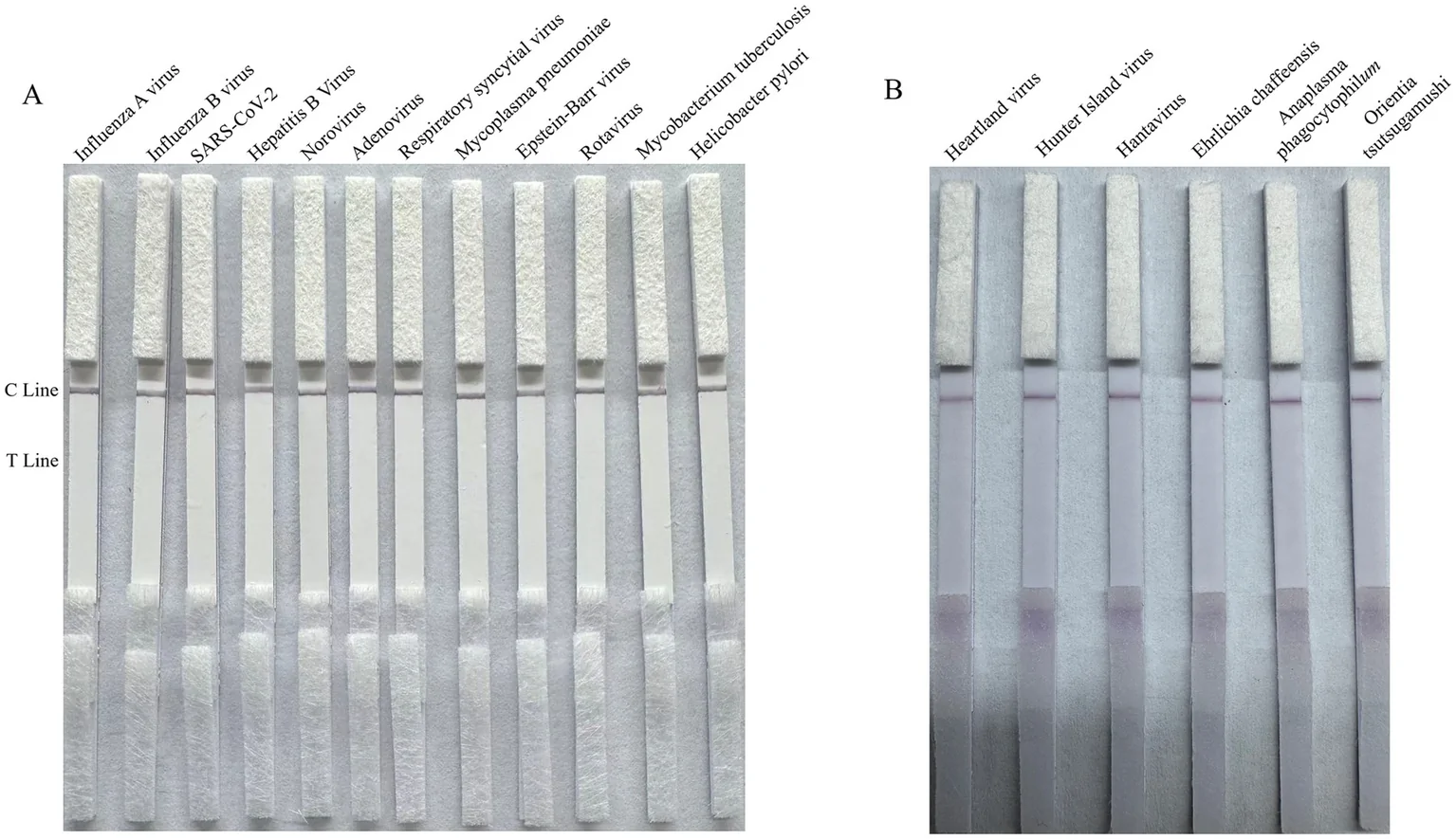
RT-RPA-LFNA cross-reactivity evaluation results. **(A)** From left to right, the samples are Influenza A virus, Influenza B virus, SARS-CoV-2, Hepatitis B virus, Norovirus, Adenovirus, Respiratory syncytial virus, *Mycoplasma pneumoniae*, Epstein–Barr virus, Rotavirus, *Mycobacterium tuberculosis*, and *Helicobacter pylori*. **(B)** From left to right, the samples are Heartland virus, Hunter Island virus, Hantavirus, *Ehrlichia chaffeensis*, *Anaplasma phagocytophilum*, *Orientia tsutsugamushi*. All test results were negative.

In this study, we collected samples from 12 clinically common pathogens distinct from SFTSV (Influenza A virus, Influenza B virus, SARS-CoV-2, Hepatitis B virus, Norovirus, Adenovirus, Respiratory syncytial virus, *Mycoplasma pneumoniae*, Epstein–Barr virus, Rotavirus, *Mycobacterium tuberculosis*, and *Helicobacter pylori*) and extracted nucleic acids. RT-RPA-LFNA validation was performed using nucleic acids from different samples as templates. The results demonstrated excellent specificity for this detection platform, with all outcomes being negative (negative result is indicated by coloration only at the C line) and no false positives detected. Specific experimental results are shown in Figure 5A. To avoid cross-reactions between this platform and other pathogens associated with SFTSV phylogenetics or showing symptoms similar to SFTSV infection (Heartland virus, Hunter Island virus, Hantavirus, *Ehrlichia chaffeensis*, *Anaplasma phagocytophilum*, *Orientia tsutsugamushi*), this study also downloaded the sequences of these pathogens from the NCBI database and synthesized corresponding cloning vectors to validate cross-reactivity. Specific experimental results are shown in Figure 5B. The results showed that the primers selected by this platform did not amplify any of these pathogens. This further confirms the absence of cross-reactivity in the platform, a critical characteristic for detecting pathogens in complex samples that effectively prevents missed diagnoses and misdiagnoses.

### Clinical sample evaluation of the RT-RPA-LFNA method

**Figure 6:**
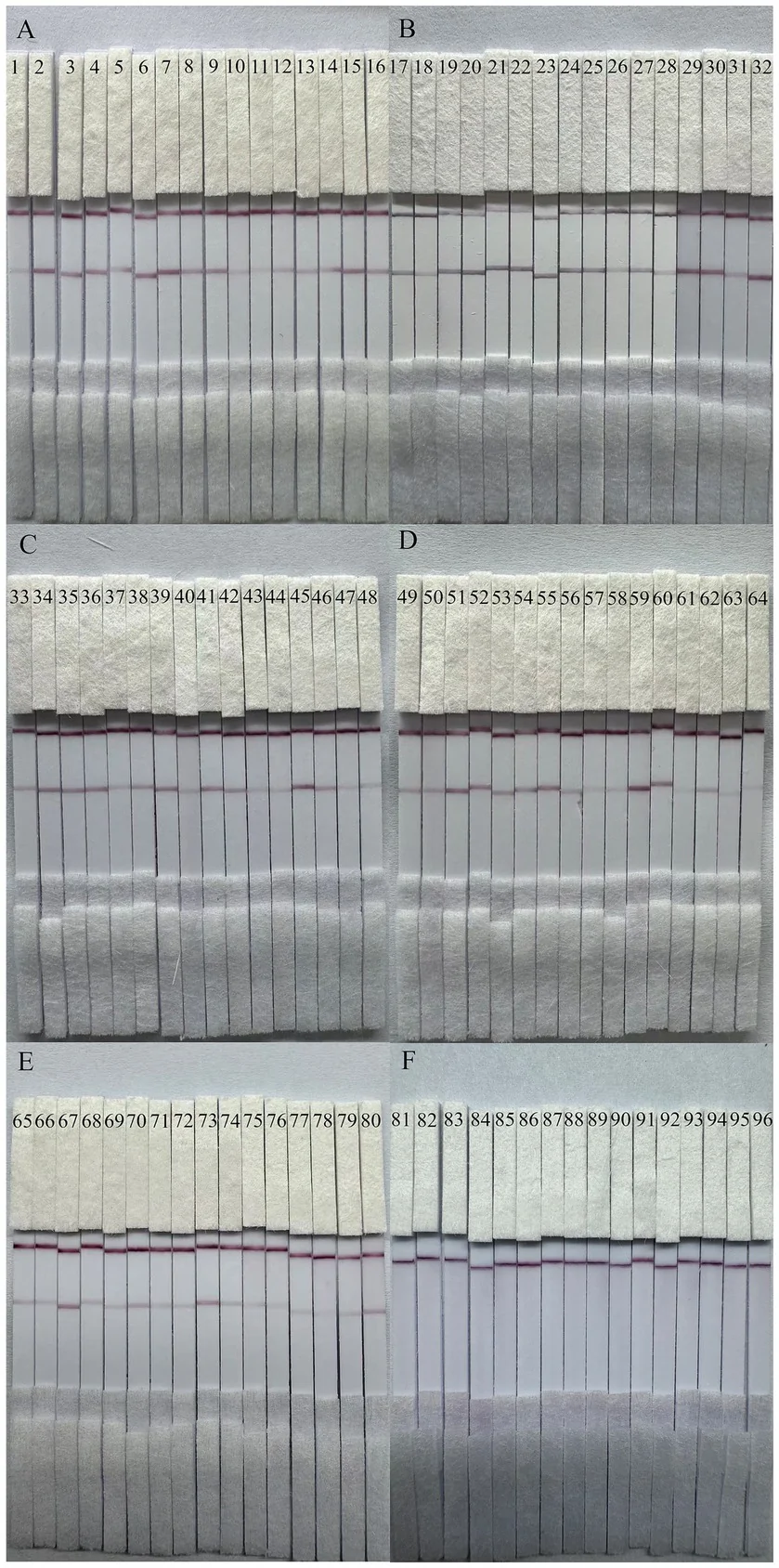
RT-RPA-LFNA clinical sample evaluation results. **(A)** Evaluation results for clinical positive samples 1–16. **(B)** Evaluation results for clinical positive samples 17–32. **(C)** Evaluation results for clinical positive samples 33–48. **(D)** Evaluation results for clinical positive samples 49–64. **(E)** Evaluation results for clinical positive samples 65–80. All 80 clinical positive samples tested positive. **(F)** Evaluation results for clinical negative samples 81–96. All 16 clinical negative samples tested negative. 95% confidence interval (Clopper-Pearson exact method): sensitivity 95% CI = [95.49, 100.00%], Specificity 95% CI = [79.41, 100.00%].

In this study, we collected 80 clinical samples confirmed positive and 16 clinical samples confirmed negative for SFTSV at the North Campus of the First Affiliated Hospital of Anhui Medical University. The viral load and Ct value of all clinical samples were determined by qPCR, the specific results are shown in Supplementary Table S4. Moreover, after nucleic acid extraction, these samples were validated using RT-RPA-LFNA. Results showed that all 80 positive samples exhibited positive reactions on the test strips. Additionally, all 16 negative samples exhibited negative reactions. This confirms the excellent sensitivity and specificity of the platform, which is capable of detecting the majority of SFTSV patients. Specific results are shown in Figure 6. We also analyzed the ratio of the grayscale values of the T line to those of the C line for all test results; the specific results are shown in Supplementary Table S4.

## Discussion

In the aforementioned experiment, we developed an RT-RPA-LFNA detection platform with a sensitivity of 10^1^ copies/μL, comparable to that of qPCR. The platform showed no cross-reactivity with common clinical pathogens or pathogens phylogenetically closely related to SFTSV, and demonstrated good specificity and sensitivity in the evaluation of 96 clinical samples. Compared with previous molecular methods for detecting SFTSV, RT-RPA-LFNA offers the advantages of simple primer design, a short turnaround time, a low limit of detection and visual readout. Specific nucleic acid tags and probes enable the test results to be interpreted intuitively on the test strip.

The novel bunyavirus, mainly transmitted by ticks and through contact with infected animals or patient body fluids, causes severe hemorrhagic fever with a high fatality rate (16–30%) (Zhou et al., 2022; Li et al., 2018) and currently lacks effective vaccines or treatments. Although qPCR remains the gold standard for detection, it requires specialized equipment, trained personnel, and is time-consuming and costly—limitations that hinder its use in primary care and resource-limited settings. Thus, developing rapid, simple, and low-cost early detection methods is crucial to reducing diagnostic delays and improving patient outcomes.

This study established a diagnostic method combining RT-RPA with LFNA, thereby integrating RT-RPA’s rapid amplification advantage with LFNA’s simple, visual colorimetric detection. Simply perform RT-RPA on the target nucleic acid and add it to LFNA; disease status can then be determined by observing color development on the test strip. Compared to the gold standard qPCR, this detection platform is simpler to operate, requiring only a metal bath for testing. Moreover, the platform is faster, requiring only 40 min to complete the detection, which significantly shortens the testing cycle and provides faster guidance for clinical diagnosis.

Our detection platform demonstrated outstanding performance, achieving excellent specificity, sensitivity, and concordance across 96 clinical samples, indicating high accuracy. In the sensitivity assessment, the LOD for the SFTSV standard clone carrier reached 10^1^ copies/μL using this method. Furthermore, no cross-reactivity was observed when testing for 12 other common pathogens, as well as other pathogens associated with SFTSV phylogenetics or showing symptoms similar to SFTSV infection. The experimental results above indicate that the RT-RPA-LFNA detection method possesses good specificity and sensitivity, has the potential for point-of-care testing (POCT) in resource-limited settings, and offers broad prospects for application.

However, we must also acknowledge the limitations of our research platform. For instance, pathogen detection using this platform requires nucleic acid extraction, which means that laboratories must be equipped with the appropriate nucleic acid extraction equipment and reagents. This is the primary limitation to the platform’s transition to POCT. Furthermore, opening the reaction vessel after RT-RPA amplification to transfer the product unavoidably increases the risk of false positives and aerosol contamination. This necessitates that POC laboratories or testing sites using this method be equipped with biosafety cabinets (BSC). Additionally, although the testing time for the platform’s test strips is short, the assembly of the strips themselves is time-consuming and challenging, the technical challenges associated with strip assembly are also part of the platform’s limitations. Moreover, as we have a limited number of negative samples, which do not fully reflect the clinical sample population, a prospective validation study involving a larger clinical sample is required. Furthermore, the fact that qPCR was used as the reference method without being tested independently is a limitation of our experiment. As we have not conducted any studies on reproducibility and robustness, the robustness of this detection platform requires further optimization. We can mitigate aerosol contamination by using filter pipette tips or optimizing the ventilation system of the biosafety cabinet, as well as by monitoring airborne nucleic acid contamination by leaving PCR tubes open containing sterile water. Additionally, new methods must be explored to overcome the limitations of nucleic acid extraction. Therefore, in future research, further expansion should be conducted to enhance the comprehensive detection capability of the platform, thereby better meeting the diverse needs of clinical and public health sectors.

In conclusion, based on RT-RPA-LFNA technology, this study developed and validated a rapid diagnostic platform for the novel Bunyavirus, providing a solution for the rapid detection of SFTSV. The platform can detect the target pathogen within 40 min, achieving simple, fast, and low-cost detection, and has the potential for use in point-of-care testing (POCT). Furthermore, this platform holds significant potential for application in the detection of other pathogens and can be further developed for widespread use in resource-limited settings, offering strong technical support for public health prevention and control.

## Data Availability

The original contributions presented in the study are included in the article/Supplementary material, further inquiries can be directed to the corresponding authors.

## References

[ref1] Bratuleanu B. E. Răileanu C. Bennouna A. Chretien D. Bigot T. Guardado-Calvo P. . (2023). Diversity of viruses in *Ixodes ricinus* in Europe including novel and potential arboviruses. Transbound. Emerg. Dis. 2023, 1–14. doi: 10.1155/2023/6661723, 40303763 PMC12017061

[ref2] Bryden S. R. Dunlop J. I. Clarke A. T. Fares M. Pingen M. Wu Y. . (2022). Exploration of immunological responses underpinning severe fever with thrombocytopenia syndrome virus infection reveals IL-6 as a therapeutic target in an immunocompromised mouse model. PNAS Nexus 1:pgac024. doi: 10.1093/pnasnexus/pgac024, 35529317 PMC9071185

[ref3] Chang Y. C. Shimoda H. Wen H. W. Maeda K. Tseng C. Y. Tsai C. H. . (2025). Enhanced detection of severe fever with thrombocytopenia syndrome virus (SFTSV) antibodies in various animal species using oligomeric nucleocapsid protein-based diagnostics. Virol. J. 22:210. doi: 10.1186/s12985-025-02840-5, 40588737 PMC12207807

[ref4] Chen Q. L. Zhu M. T. Chen N. Yang D. Yin W. W. Mu D. . (2022). Epidemiological characteristics of severe fever with thtrombocytopenia syndrome in China, 2011-2021. Zhonghua Liu Xing Bing Xue Za Zhi 43, 852–859. doi: 10.3760/cma.j.cn112338-20220325-00228, 35725341

[ref5] Cui H. Shen S. Chen L. Fan Z. Wen Q. Xing Y. . (2024). Global epidemiology of severe fever with thrombocytopenia syndrome virus in human and animals: a systematic review and meta-analysis. Lancet Reg Health West Pac. 48:101133. doi: 10.1016/j.lanwpc.2024.101133, 39040038 PMC11261768

[ref6] Fan Y. Wang J. Bian X. Wang W. Zhou J. Zhang X. . (2025). Multienzyme isothermal rapid amplification (MIRA)-LFD assay for rapid detection of PEDV and PoRVA. J. Microbiol. Methods 238:107289. doi: 10.1016/j.mimet.2025.107289, 41076143

[ref7] Gao S. Geng X. Lu Q. Wu S. Shan Z. Chang C. (2023). Epidemiological characteristics and spatio-temporal aggregation of severe fever with thrombocytopenia syndrome in Jinan City, China, 2018–2022. PLoS Negl. Trop. Dis. 17:e0011807. doi: 10.1371/journal.pntd.0011807, 38134002 PMC10745217

[ref8] Goldsmith C. S. Ksiazek T. G. Rollin P. E. Comer J. A. Nicholson W. L. Peret T. C. T. . (2013). Cell culture and Electron microscopy for identifying viruses in diseases of unknown cause. Emerg. Infect. Dis. 19, 864–869. doi: 10.3201/eid1906.130173, 23731788 PMC3713842

[ref9] Huang M. Liu S. Xu Y. Li A. Wu W. Liang M. . (2022). CRISPR/Cas12a technology combined with RPA for rapid and portable SFTSV detection. Front. Microbiol. 13. 10.3389/fmicb.2022.754995 PMC8822122 35145502

[ref10] Jiao Y. Zeng X. Guo X. Qi X. Zhang X. Shi Z. . (2012). Preparation and evaluation of recombinant severe fever with thrombocytopenia syndrome virus Nucleocapsid protein for detection of Total antibodies in human and animal sera by double-antigen Sandwich enzyme-linked immunosorbent assay. J. Clin. Microbiol. 50, 372–377. doi: 10.1128/JCM.01319-11, 22135253 PMC3264160

[ref11] Lee B. Park B. Kim D. Jung C. Park J. H. Park J.-H. . (2025). Lateral flow immunoassay using plasmonic scattering. Nat. Commun. 16:3377. doi: 10.1038/s41467-025-58663-z, 40204766 PMC11982407

[ref12] Lee J. W. Won Y. J. Kang L. H. Lee S. G. Park S. W. Paik S. Y. (2020). Development of a real-time loop-mediated isothermal amplification method for the detection of severe fever with thrombocytopenia syndrome virus. J. Microbiol. 58, 711–715. doi: 10.1007/s12275-020-0109-1, 32424580 PMC7232587

[ref13] Li J. Kelly P. Guo W. Zhang J. Yang Y. Liu W. . (2020). Molecular detection of *Rickettsia*, *Hepatozoon*, *Ehrlichia* and SFTSV in goat ticks. Vet. Parasitol. 20:100407. doi: 10.1016/j.vprsr.2020.100407 32448525

[ref14] Li J. Li S. Yang L. Cao P. Lu J. (2021). Severe fever with thrombocytopenia syndrome virus: a highly lethal bunyavirus. Crit. Rev. Microbiol. 47, 112–125. doi: 10.1080/1040841X.2020.1847037, 33245676

[ref15] Li A. Liu L. Wu W. Liu Y. Huang X. Li C. . (2021). Molecular evolution and genetic diversity analysis of SFTS virus based on next-generation sequencing. Biosaf. Health 3, 105–115. doi: 10.1016/j.bsheal.2021.02.002

[ref16] Li H. Lu Q.-B. Xing B. Zhang S.-F. Liu K. Du J. . (2018). Epidemiological and clinical features of laboratory-diagnosed severe fever with thrombocytopenia syndrome in China, 2011–17: a prospective observational study. Lancet Infect. Dis. 18, 1127–1137. doi: 10.1016/S1473-3099(18)30293-7, 30054190

[ref17] Majdinasab M. Lamy de la Chapelle M. Marty J. L. (2023). Recent progresses in optical biosensors for interleukin 6 detection. Biosensors 13:898. doi: 10.3390/bios13090898, 37754132 PMC10526799

[ref18] Park J.-Y. Sivasankar C. Kirthika P. Prabhu D. Lee J. H. (2023). Non-structural protein-W61 as a novel target in severe fever with thrombocytopenia syndrome virus (SFTSV): an in-vitro and in-silico study on protein-protein interactions with nucleoprotein and viral replication. Viruses 15:1963. doi: 10.3390/v15091963, 37766369 PMC10535573

[ref19] Piepenburg O. Williams C. H. Stemple D. L. Armes N. A. (2006). DNA detection using recombination proteins. PLoS Biol. 4:e204. doi: 10.1371/journal.pbio.0040204, 16756388 PMC1475771

[ref20] Prajapati A. Tandon A. Nain V. (2022). Towards the diagnosis of dengue virus and its serotypes using designed CRISPR/Cas13 gRNAs. Bioinformation 18, 661–668. doi: 10.6026/97320630018661, 37323556 PMC10266368

[ref21] Prayitno S. P. Cho Y. G. Kim E. S. Park K. Lee S. Natasha A. . (2025). Amplicon-based MinION sequencing complements severe fever with thrombocytopenia syndrome (SFTS) diagnosis via real-time RT-PCR in patients with suspected SFTS. J. Korean Med. Sci. 40:e69. doi: 10.3346/jkms.2025.40.e69, 40390583 PMC12089687

[ref22] Qin P. Xu J. Yao L. Wu Q. Yan C. Lu J. . (2021). Simultaneous and accurate visual identification of chicken, duck and pork components with the molecular amplification integrated lateral flow strip. Food Chem. 339:127891. doi: 10.1016/j.foodchem.2020.127891, 32861930

[ref23] Reece L. M. Beasley D. W. C. Milligan G. N. Sarathy V. V. Barrett A. D. T. (2018). Current status of severe fever with thrombocytopenia syndrome vaccine development. Curr. Opin. Virol. 29, 72–78. doi: 10.1016/j.coviro.2018.03.005, 29642053

[ref24] Sun Y. Liang M. Qu J. Jin C. Zhang Q. Li J. . (2012). Early diagnosis of novel SFTS bunyavirus infection by quantitative real-time RT-PCR assay. J. Clin. Virol. 53, 48–53. doi: 10.1016/j.jcv.2011.09.031, 22024488

[ref25] Tan M. Liao C. Liang L. Yi X. Zhou Z. Wei G. (2022). Recent advances in recombinase polymerase amplification: principle, advantages, disadvantages and applications. Front. Cell. Infect. Microbiol. 12:1019071. doi: 10.3389/fcimb.2022.1019071, 36519130 PMC9742450

[ref26] Varghese J. De Silva I. Millar D. S. (2023). Latest advances in arbovirus diagnostics. Microorganisms 11:1159. doi: 10.3390/microorganisms11051159, 37317133 PMC10223626

[ref27] Vogel D. Thorkelsson S. R. Quemin E. R. J. Meier K. Kouba T. Gogrefe N. . (2020). Structural and functional characterization of the severe fever with thrombocytopenia syndrome virus L protein. Nucleic Acids Res. 48, 5749–5765. doi: 10.1093/nar/gkaa253, 32313945 PMC7261188

[ref28] Wang S. Li J. Niu G. Wang X. Ding S. Jiang X. . (2015). SFTS virus in ticks in an endemic area of China. Am. Soc. Trop. Med. Hyg. 92, 684–689. doi: 10.4269/ajtmh.14-0008, 25711611 PMC4385759

[ref29] Wang X. Zhang Q. Hao F. Gao X. Wu W. Liang M. . (2014). Development of a colloidal gold kit for the diagnosis of severe fever with thrombocytopenia syndrome virus infection. Biomed. Res. Int. 2014:530621. doi: 10.1155/2014/530621, 24826381 PMC4009110

[ref30] Wei Y. Ding S. Chen G. Dong J. Du F. Huang X. . (2022). Real-time fluorescence and colorimetric identification of bulbus fritillariae using recombinase assisted loop-mediated isothermal DNA amplification (RALA). Front. Plant Sci. 13. doi: 10.3389/fpls.2022.948879, 35968097 PMC9366889

[ref31] Wen Y. Fang Y. Cao F. Zhang G. Cheng S. Yu Y. . (2024). A person-to-person transmission cluster of severe fever with thrombocytopenia syndrome characterized by mixed viral infections with familial and nosocomial clustering. Heliyon. 10:e24502. doi: 10.1016/j.heliyon.2024.e24502, 38298613 PMC10827760

[ref32] Wu W. Biyani M. Hirose D. Takamura Y. (2023). Rapid and highly sensitive detection of Leishmania by combining recombinase polymerase amplification and solution-processed oxide thin-film transistor technology. Biosensors 13:765. doi: 10.3390/bios13080765, 37622851 PMC10452724

[ref33] Zhang M. Du Y. Yang L. Zhan L. Yang B. Huang X. . (2022). Development of monoclonal antibody based IgG and IgM ELISA for diagnosis of severe fever with thrombocytopenia syndrome virus infection. Braz. J. Infect. Dis. 26:102386. doi: 10.1016/j.bjid.2022.102386, 35835158 PMC9459026

[ref34] Zhou C.-M. Qi R. Qin X.-R. Fang L.-Z. Han H.-J. Lei X.-Y. . (2022). Oral and ocular transmission of severe fever with thrombocytopenia syndrome virus. Inf. Med. 1, 2–6. doi: 10.1016/j.imj.2021.12.002, 38074978 PMC10699656

[ref35] Zhu Y. Zhang M. Jie Z. Tao S. (2022). Nucleic acid testing of SARS-CoV-2: a review of current methods, challenges, and prospects. Front. Microbiol. 13:1074289. doi: 10.3389/fmicb.2022.1074289 PMC9780671 36569096

[ref36] Zhuang L. Sun Y. Cui X. M. Tang F. Hu J. G. Wang L. Y. . (2018). Transmission of severe fever with thrombocytopenia syndrome virus by *Haemaphysalis longicornis* ticks, China. Emerg. Infect. Dis. 24, 868–871. doi: 10.3201/eid2405.151435, 29664718 PMC5938789

