# Supplementary material for: Establishment of a rapid detection method for severe fever with thrombocytopenia syndrome virus based on reverse transcription-recombinase polymerase amplification with lateral flow nucleic acid chromatography assay technology
Source: Front Microbiol. 2026 Sep 9;17:1934648. doi: 10.3389/fmicb.2026.1934648 (PMC13597815; doi:10.3389/fmicb.2026.1934648)
Supplement: Supplementary file 1 [file Table_1.docx]

Table S1 Epidemiological information of the 80 positive clinical samples

| Sample number | Date | Age | Region |
| --- | --- | --- | --- |
| 1 | 4.6 | 70-79 | Anqing |
| 2 | 4.18 | 70-79 | Hefei |
| 3 | 4.25 | 70-79 | Hefei |
| 4 | 4.30 | 50-60 | Hefei |
| 5 | 4.30 | 60-70 | Lu´an |
| 6 | 5.1 | 70-79 | Lu´an |
| 7 | 5.2 | 50-52 | Chuzhou |
| 8 | 5.2 | 50-59 | Maanshan |
| 9 | 5.4 | 60-69 | Hefei |
| 10 | 5.4 | 50-59 | Hefei |
| 11 | 5.5 | 70-79 | Hefei |
| 12 | 5.6 | 50-59 | Anqing |
| 13 | 5.7 | 60-69 | Hefei |
| 14 | 5.8 | 70-79 | Hefei |
| 15 | 5.9 | 50-59 | Maanshan |
| 16 | 5.10 | ＜50 | Luan |
| 17 | 5.10 | 70-79 | Hefei |
| 18 | 5.11 | 50-59 | Hefei |
| 19 | 5.14 | 70-79 | Lu´an |
| 20 | 5.16 | 50-59 | Hefei |
| 21 | 5.17 | 70-79 | Hefei |
| 22 | 5.20 | 70-79 | Lu´an |
| 23 | 5.21 | 70-79 | Hefei |
| 24 | 5.22 | 70-79 | Hefei |
| 25 | 5.23 | 70-79 | Hefei |
| 26 | 5.25 | 60-69 | Anqing |
| 27 | 5.28 | ≥80 | Hefei |
| 28 | 5.29 | 50-59 | Tongling |
| 29 | 5.30 | 70-79 | Hefei |
| 30 | 6.3 | ≥80 | Anqing |
| 31 | 6.5 | 70-79 | Hefei |
| 32 | 6.7 | 70-79 | Huainan |
| 33 | 6.8 | 50-59 | Chuzhou |
| 34 | 6.10 | ≥80 | Hefei |
| 35 | 6.11 | 70-79 | Anqing |
| 36 | 6.13 | ≥80 | Lu´an |
| 37 | 6.15 | 70-79 | Hefei |
| 38 | 6.16 | 50-59 | Haozhou |
| 39 | 6.17 | ＜50 | Anqing |
| 40 | 6.19 | 50-59 | Hefei |
| 41 | 6.19 | 70-79 | Bengbu |
| 42 | 6.23 | 60-69 | Hefei |
| 43 | 6.24 | 50-59 | Hefei |
| 44 | 6.25 | ≥80 | Luan |
| 45 | 6.27 | 60-69 | Hefei |
| 46 | 6.28 | 50-59 | Hefei |
| 47 | 6.29 | 70-79 | Hefei |
| 48 | 6.30 | ≥80 | Hefei |
| 49 | 7.3 | 50-59 | Lu´an |
| 50 | 7.4 | 70-79 | Hefei |
| 51 | 7.4 | ≥80 | Hefei |
| 52 | 7.5 | 60-69 | Anqing |
| 53 | 7.8 | 60-69 | Luan |
| 54 | 7.8 | 60-69 | Hefei |
| 55 | 7.11 | 50-59 | Hefei |
| 56 | 7.13 | 60-69 | Hefei |
| 57 | 7.14 | 50-59 | Hefei |
| 58 | 7.14 | 60-69 | Hefei |
| 59 | 7.18 | 50-59 | Anqing |
| 60 | 7.20 | 70-79 | Hefei |
| 61 | 7.24 | 50-59 | Hefei |
| 62 | 7.27 | 70-79 | Lu´an |
| 63 | 8.2 | 60-69 | Hefei |
| 64 | 8.3 | 60-69 | Hefei |
| 65 | 8.8 | 70-79 | Hefei |
| 66 | 8.13 | 60-69 | Hefei |
| 67 | 8.18 | 50-59 | Hefei |
| 68 | 8.21 | 70-79 | Anqing |
| 69 | 9.22 | 60-69 | Fuyang |
| 70 | 10.2 | 60-69 | Anqing |
| 71 | 10.3 | 70-79 | Luan |
| 72 | 10.6 | 70-79 | Hefei |
| 73 | 10.10 | ≥80 | Hefei |
| 74 | 10.11 | 70-79 | Hefei |
| 75 | 10.15 | 50-59 | Hefei |
| 76 | 10.18 | 70-79 | Hefei |
| 77 | 10.21 | 70-79 | Hefei |
| 78 | 10.26 | 70-79 | Hefei |
| 79 | 10.27 | 70-79 | Lu´an |
| 80 | 11.7 | ≥80 | Hefei |

Table S2 Nucleic acid concentration and purity of positive samples 1-80

| Sample number | Nucleic acid concentration（ng/μL） | A260/A280 |
| --- | --- | --- |
| 1 | 6.81 | 1.66 |
| 2 | 56.30 | 1.87 |
| 3 | 18.36 | 1.50, |
| 4 | 21.61 | 1.58 |
| 5 | 13.81 | 1.58 |
| 6 | 20.19 | 1.59 |
| 7 | 28.16 | 1.52 |
| 8 | 17.13 | 1.59 |
| 9 | 18.87 | 1.50 |
| 10 | 5.81 | 1.72 |
| 11 | 5.62 | 1.59 |
| 12 | 7.57 | 1.42 |
| 13 | 8.68 | 1.55 |
| 14 | 12.22 | 1.52 |
| 15 | 23.43 | 1.55 |
| 16 | 7.69 | 1.68 |
| 17 | 9.84 | 1.50 |
| 18 | 6.04 | 1.48 |
| 19 | 10.55 | 1.47 |
| 20 | 17.60 | 1.52 |
| 21 | 18.78 | 1.49 |
| 22 | 35.35 | 1.47 |
| 23 | 13.61 | 1.48 |
| 24 | 13.30 | 1.54 |
| 25 | 16.78 | 1.52 |
| 26 | 8.61 | 1.47 |
| 27 | 7.69 | 1.68 |
| 28 | 3.54 | 1.57 |
| 29 | 23.30 | 1.46 |
| 30 | 14.42 | 1.36 |
| 31 | 8.98 | 1.40 |
| 32 | 41.88 | 1.50 |
| 33 | 12.60 | 1.48 |
| 34 | 19.23 | 1.60 |
| 35 | 15.85 | 1.43 |
| 36 | 17.42 | 1.50 |
| 37 | 11.77 | 1.32 |
| 38 | 15.03 | 1.43 |
| 39 | 23.11 | 1.48 |
| 40 | 12.40 | 1.51 |
| 41 | 28.03 | 1.68 |
| 42 | 14.64 | 1.40 |
| 43 | 11.65 | 1.50 |
| 44 | 5.72 | 1.51 |
| 45 | 30.70 | 1.81 |
| 46 | 15.71 | 1.48 |
| 47 | 5.62 | 1.54 |
| 48 | 10.63 | 1.39 |
| 49 | 11.51 | 1.47 |
| 50 | 13.35 | 1.48 |
| 51 | 25.75 | 1.64 |
| 52 | 23.11 | 1.39 |
| 53 | 17.00 | 1.52 |
| 54 | 22.27 | 1.47 |
| 55 | 24.75 | 1.49 |
| 56 | 10.33 | 1.41 |
| 57 | 9.33 | 1.39 |
| 58 | 10.08 | 1.44 |
| 59 | 52.65 | 1.84 |
| 60 | 23.43 | 1.55 |
| 61 | 7.42 | 1.36 |
| 62 | 15.36 | 1.47 |
| 63 | 8.95 | 1.56 |
| 64 | 8.94 | 1.55 |
| 65 | 8.68 | 1.55 |
| 66 | 5.48 | 1.51 |
| 67 | 22.90 | 1.44 |
| 68 | 18.87 | 1.50 |
| 69 | 16.59 | 1.53 |
| 70 | 19.66 | 1.49 |
| 71 | 8.40 | 1.63 |
| 72 | 5.90 | 1.39 |
| 73 | 20.19 | 1.59 |
| 74 | 5.08 | 1.41 |
| 75 | 14.04 | 1.48 |
| 76 | 5.09 | 1.58 |
| 77 | 6.81 | 1.66 |
| 78 | 1.01 | 2.04 |
| 79 | 3.96 | 1.59 |
| 80 | 19.90 | 1.55 |

Table S3 Evaluation of the grayscale values for the RPA-LFNA limit of detection

| Experiment title | Sample | T-line grayscale value(pixels) | C-line grayscale value(pixels) | T/C |
| --- | --- | --- | --- | --- |
| First experiment | 10^8^copies/μL | 464944 | 402115 | 1.156246 |
|  | 10^7^copies/μL | 443636 | 492602 | 0.900597 |
|  | 10^6^copies/μL | 210025 | 441271 | 0.475955 |
|  | 10^5^copies/μL | 184063 | 435504 | 0.422644 |
|  | 10^4^copies/μL | 153902 | 392630 | 0.391977 |
|  | 10^3^copies/μL | 122711 | 387326 | 0.316816 |
|  | 10^2^copies/μL | 104892 | 598373 | 0.175295 |
|  | 10^1^copies/μL | 68541 | 554423 | 0.123626 |
|  | 10^0^copies/μL | 36545 | 491920 | 0.074291 |
|  | negative control | 33684 | 487682 | 0.06907 |
| Second experiment | 10^8^copies/μL | 582594 | 427787 | 1.361879 |
|  | 10^7^copies/μL | 537700 | 477129 | 1.126949 |
|  | 10^6^copies/μL | 401711 | 539803 | 0.744181 |
|  | 10^5^copies/μL | 318996 | 541181 | 0.589444 |
|  | 10^4^copies/μL | 209436 | 521202 | 0.401833 |
|  | 10^3^copies/μL | 184388 | 512316 | 0.359911 |
|  | 10^2^copies/μL | 111712 | 607303 | 0.183948 |
|  | 10^1^copies/μL | 79489 | 598651 | 0.13278 |
|  | 10^0^copies/μL | 37019 | 600337 | 0.061664 |
|  | negative control | 32224 | 615789 | 0.05233 |
| Third experiment | 10^8^copies/μL | 344573 | 269317 | 1.279433 |
|  | 10^7^copies/μL | 347604 | 380423 | 0.91373 |
|  | 10^6^copies/μL | 145002 | 240530 | 0.602844 |
|  | 10^5^copies/μL | 138599 | 364105 | 0.380657 |
|  | 10^4^copies/μL | 115692 | 330767 | 0.349769 |
|  | 10^3^copies/μL | 67519 | 309134 | 0.218413 |
|  | 10^2^copies/μL | 66901 | 500371 | 0.133703 |
|  | 10^1^copies/μL | 57157 | 492668 | 0.116015 |
|  | 10^0^copies/μL | 31804 | 498506 | 0.066584 |
|  | negative control | 28977 | 508570 | 0.059459 |

Table S4 Specific test results for clinical samples 1-96

| Sample number | Viral load (TCID_50_/mL) | Ct value | Copy count (copies/μL) | T/C |
| --- | --- | --- | --- | --- |
| 1 | 60.94 | 29.69 | 30.23 | 0.226842 |
| 2 | 4.07E5 | 20.23 | 2.02×10^5^ | 0.504125 |
| 3 | 1.9E3 | 26.62 | 9.42×10^2^ | 0.347361 |
| 4 | 1.92E3 | 25.52 | 9.52×10^2^ | 0.354648 |
| 5 | 122.8 | 29.41 | 60.91 | 0.203776 |
| 6 | 2.2E4 | 21.5 | 1.09×10^4^ | 0.462068 |
| 7 | 1.7E5 | 19.48 | 8.43×10^4^ | 0.616248 |
| 8 | 1.05E3 | 26.45 | 5.21×10^2^ | 0.395776 |
| 9 | 3.95E3 | 24.49 | 1.96×10^3^ | 0.431742 |
| 10 | 42.7 | 30.93 | 21.18 | 0.2329 |
| 11 | 15.6 | 32.65 | 7.74 | 0.160926 |
| 12 | 101.76 | 29.77 | 50.47 | 0.184449 |
| 13 | 251.52 | 28.63 | 124.75 | 0.299242 |
| 14 | 527.78 | 27.32 | 261.78 | 0.342846 |
| 15 | 4.42E4 | 20.96 | 2.19×10^4^ | 0.496455 |
| 16 | 227.25 | 28.58 | 112.72 | 0.280679 |
| 17 | 492.21 | 27.81 | 244.14 | 0.355134 |
| 18 | 37.79 | 30.77 | 18.74 | 0.205952 |
| 19 | 8.86E3 | 23.2 | 4.39×10^3^ | 0.420293 |
| 20 | 4.83E5 | 18.34 | 2.40×10^5^ | 0.693545 |
| 21 | 1.25E6 | 16.41 | 6.20×10^5^ | 0.973481 |
| 22 | 1.81E7 | 14.41 | 8.98×10^6^ | 1.146327 |
| 23 | 1.12E4 | 24.96 | 5.56×10^3^ | 0.550188 |
| 24 | 9.65E3 | 25.48 | 4.79×10^3^ | 0.494184 |
| 25 | 2.36E3 | 25.41 | 1.17×10^3^ | 0.386652 |
| 26 | 53.51 | 28.08 | 26.54 | 0.245255 |
| 27 | 228.93 | 27.99 | 113.55 | 0.332588 |
| 28 | 37.06 | 30.79 | 18.38 | 0.237505 |
| 29 | 2.15E6 | 15.64 | 1.07×10^6^ | 1.08995 |
| 30 | 1.81E4 | 22.39 | 8.98×10^3^ | 0.621494 |
| 31 | 83 | 31.58 | 41.17 | 0.285348 |
| 32 | 1.04E8 | 10.02 | 5.16×10^7^ | 1.299002 |
| 33 | 492.72 | 28.04 | 244.39 | 0.195312 |
| 34 | 7.79E3 | 23.62 | 3.86×10^3^ | 0.411763 |
| 35 | 3.43E3 | 24.24 | 1.70×10^3^ | 0.358098 |
| 36 | 6.33E3 | 23.78 | 3.14×10^3^ | 0.387895 |
| 37 | 290 | 28.41 | 143.84 | 0.173657 |
| 38 | 1.09E3 | 27.57 | 5.41×10^2^ | 0.326186 |
| 39 | 5.16E4 | 20.65 | 2.56×10^4^ | 0.456003 |
| 40 | 221.59 | 29.51 | 109.91 | 0.159011 |
| 41 | 7.63E4 | 21.04 | 3.78×10^4^ | 0.488239 |
| 42 | 1.05E3 | 28.17 | 5.21×10^2^ | 0.307849 |
| 43 | 166 | 29.9 | 82.34 | 0.16551 |
| 44 | 65.89 | 30.1 | 32.68 | 0.139097 |
| 45 | 2.01E6 | 14.38 | 9.97×10^5^ | 0.817372 |
| 46 | 1.33E3 | 25.42 | 6.60×10^2^ | 0.384974 |
| 47 | 72.98 | 30.26 | 36.20 | 0.142189 |
| 48 | 103.73 | 29.27 | 51.45 | 0.153167 |
| 49 | 1.74E3 | 25.11 | 8.63×10^2^ | 0.329001 |
| 50 | 3.42E3 | 24.69 | 1.70×10^3^ | 0.378982 |
| 51 | 4.6E5 | 19.32 | 2.28×10^5^ | 0.583832 |
| 52 | 2.26E5 | 20.21 | 1.12×10^5^ | 0.523721 |
| 53 | 1.46E4 | 23.49 | 7.24×10^3^ | 0.433394 |
| 54 | 3.78E4 | 22.81 | 1.87×10^4^ | 0.449092 |
| 55 | 6.03E4 | 21.47 | 2.99×10^4^ | 0.478687 |
| 56 | 654.18 | 28.72 | 324.47 | 0.242056 |
| 57 | 153.6 | 29.39 | 76.19 | 0.179604 |
| 58 | 167.18 | 29.46 | 82.92 | 0.180243 |
| 59 | 6.01E7 | 10.44 | 2.99×10^7^ | 1.007957 |
| 60 | 4.0E5 | 19.88 | 1.98×10^5^ | 0.577291 |
| 61 | 11.06 | 31.64 | 5.49 | 0.133219 |
| 62 | 4.18E3 | 24.93 | 2.07×10^3^ | 0.379308 |
| 63 | 102.43 | 28.8 | 50.81 | 0.169395 |
| 64 | 53.56 | 30.89 | 26.57 | 0.136701 |
| 65 | 486.13 | 27.75 | 241.12 | 0.243124 |
| 66 | 127.52 | 29.01 | 63.25 | 0.201043 |
| 67 | 1.15E5 | 20.43 | 5.70×10^4^ | 0.436884 |
| 68 | 744.26 | 27.49 | 369.15 | 0.28908 |
| 69 | 479.79 | 27.66 | 237.98 | 0.239086 |
| 70 | 1.01E3 | 26.37 | 5.01×10^2^ | 0.303977 |
| 71 | 189.64 | 28.61 | 95.06 | 0.212031 |
| 72 | 84.61 | 29.13 | 41.97 | 0.194164 |
| 73 | 6.03E4 | 22.77 | 2.99×10^4^ | 0.425507 |
| 74 | 109 | 28.82 | 54.06 | 0.19639 |
| 75 | 526 | 27.58 | 260.90 | 0.268968 |
| 76 | 74.38 | 29.02 | 36.89 | 0.193032 |
| 77 | 118.47 | 27.4 | 58.76 | 0.202322 |
| 78 | 3.97 | 34.49 | 1.97 | 0.104058 |
| 79 | 43.69 | 30.33 | 21.67 | 0.150905 |
| 80 | 1.16E4 | 23.61 | 5.75×10^3^ | 0.40846 |
| 81 | Undetermined | |  | 0.058967 |
| 82 | Undetermined | |  | 0.063436 |
| 83 | Undetermined | |  | 0.078633 |
| 84 | Undetermined | |  | 0.069429 |
| 85 | Undetermined | |  | 0.070386 |
| 86 | Undetermined | |  | 0.052327 |
| 87 | Undetermined | |  | 0.075676 |
| 88 | Undetermined | |  | 0.06742 |
| 89 | Undetermined | |  | 0.065908 |
| 90 | Undetermined | |  | 0.072152 |
| 91 | Undetermined | |  | 0.065293 |
| 92 | Undetermined | |  | 0.046021 |
| 93 | Undetermined  Undetermined  Undetermined  Undetermined | |  | 0.055665 |
| 94 |  |  |  | 0.061946 |
| 95 |  |  |  | 0.057046 |
| 96 |  |  |  | 0.051874 |
